# Overexpression of miR-21-5p as a predictive marker for complete tumor regression to neoadjuvant chemoradiotherapy in rectal cancer patients

**DOI:** 10.1186/s12920-014-0068-7

**Published:** 2014-12-11

**Authors:** Camila Miranda Lopes-Ramos, Angelita Habr-Gama, Bruna de Souza Quevedo, Natália Mariana Felício, Fabiana Bettoni, Fernanda Christtanini Koyama, Paula Fontes Asprino, Pedro Alexandre Galante, Joaquim Gama-Rodrigues, Anamaria Aranha Camargo, Rodrigo Oliva Perez, Raphael Bessa Parmigiani

**Affiliations:** Centro de Oncologia Molecular, Hospital Sírio-Libanês, São Paulo, Brazil; Fundação Antônio Prudente, São Paulo, Brazil; Angelita & Joaquim Gama Institute, São Paulo, Brazil; University of São Paulo School of Medicine, São Paulo, Brazil; Ludwig Institute for Cancer Research, São Paulo, Brazil; Hospital Alemão Oswaldo Cruz, São Paulo, Brazil

**Keywords:** miRNA, Predictive biomarker, Rectal cancer, miR-21-5p, SATB1, Chemoradiotherapy

## Abstract

**Background:**

Neoadjuvant chemoradiotherapy (nCRT) followed by radical surgery is the preferred treatment strategy for locally advanced rectal cancer. However, complete tumor regression is observed in a significant proportion of patients after nCRT, making them ideal candidates for alternative treatment strategies to this considerably morbid procedure. Identification of such patients based on clinical findings (complete clinical response - cCR) is difficult mainly because it relies on subjective clinical and imaging studies. Our goal was to identify biomarkers capable of predicting complete response to nCRT.

**Methods:**

We analyzed miRNA expression profile using deep sequencing in rectal tumor biopsies prior to nCRT. Differential expression was investigated by EdgeR for a training (n = 27) and a validation (n = 16) set of patients to identify miRNAs associated with treatment response (complete vs. incomplete). *In vitro* experiments with two cancer cell lines were also performed in order to evaluate the possible role of miRNAs on response to nCRT.

**Results:**

We found 4 miRNAs differentially expressed between complete and incomplete responders to nCRT. In addition, validation was performed using an independent group of patients and miR-21-5p was confirmed as being overexpressed in complete responders. Overall sensitivity and specificity of miR-21-5p expression in predicting complete response to nCRT was 78% and 86% respectively. Interestingly, in a subset of patients with cCR followed by early local recurrence, the expression level of miR-21-5p was considerably low, similarly to incomplete responders. We also found SATB1, a miR-21-5p target gene and known multidrug resistance gene, whose expression was inversely correlated with miR-21-5p expression. Finally, we performed functional experiments and showed that miR-21-5p and SATB1 may be directly involved with poor response to nCRT in rectal cancer patients.

**Conclusions:**

This study suggests miR-21-5p as a promising predictive biomarker, which should aid in the selection of patients with cCR to nCRT that potentially could be spared from radical surgery.

**Electronic supplementary material:**

The online version of this article (doi:10.1186/s12920-014-0068-7) contains supplementary material, which is available to authorized users.

## Background

Treatment of locally advanced rectal cancer includes a combination of surgery and radiotherapy with or without chemotherapy (CRT and RT, respectively). In recent years, preoperative delivery of RT or CRT has become one of the preferred initial treatment options for the management of rectal cancer due to improved local disease control and significant tumor regression [1-3].

Tumor regression to neoadjuvant chemoradiation (nCRT) varies substantially among patients, and ultimately, complete pathological response (pCR) may develop in up to 42% of them [4]. These patients with pCR are associated with excellent oncological outcomes, particularly in terms of local disease control. Considering that radical surgery does not remove any residual cancer in these patients, alternative treatment strategies to this procedure have been considered in an effort to avoid unnecessary postoperative morbidity and mortality. A strategy of close observation without immediate surgery, known as “Watch and Wait”, has been proposed for highly selected patients with no clinically or radiologically detectable residual tumor (complete clinical response – cCR) [5]. This strategy requires an intensive follow-up in which patients are submitted to frequent clinical, endoscopic and radiological assessments after completing nCRT [6].

However, clinical and radiological assessment of tumor response remains a significant challenge due to their subjectivity and inherent limitations of currently available studies. In this setting, identification of molecular markers capable of predicting complete response to nCRT would not only allow accurate selection of patients that benefit the most from nCRT but also identify ideal candidates to alternative treatment strategies without immediate radical surgery after achieving cCR.

miRNAs are small non-coding RNAs (18-25 nt) capable of regulating mRNAs post-transcriptionally by inducing their destabilization or translational repression [7]. miRNAs play important regulatory roles in several cellular processes such as cell proliferation, differentiation, and apoptosis. Changes in their expression profile have been reported for several types of cancer [8]. In fact, miRNAs may contribute to the tumorigenic process functioning as tumor suppressor genes or oncogenes depending on the genes they regulate [9,10].

Importantly, different studies have also investigated how miRNA profiling may be associated with treatment response in different tumor types, motivating the search for their role as predictive molecular markers in cancer [11-13]. The goal of the present study was to compare miRNA expression profile of treatment-naive tumor biopsies derived from rectal cancer patients with complete or incomplete response to nCRT in order to identify specific miRNAs as predictive biomarkers.

## Methods

### Patients and biological samples

Patients with biopsy-proven rectal adenocarcinoma, cT2-4 N0-2 M0 located no more than 7 cm from the anal verge measured by rigid proctoscopy were eligible for the study. All patients were radiologically staged using high-resolution pelvic MRI, abdominal and chest CT scans and CEA levels. Patients with pregnancy or under the age of 18 were excluded from the study.

Endoscopic biopsies were taken from primary tumors, properly identified and snap frozen using liquid nitrogen prior to storage at −80°C. Tumor fragments were verified for the presence of ≥80% viable cancer cells prior to RNA extraction using standard hematoxilin-eosin staining. Specimens with <80% cancer cells were macro-dissected to result in ≥80% of cancer cells.

This study was approved by the ethics committee from Hospital Alemão Oswaldo Cruz (Sao Paulo, Brazil - reference number 19/08). Informed consent was obtained from all study participants prior to sample collection.

### Treatment and assessment of response

All patients underwent nCRT as described elsewhere [5]. Briefly, nCRT consisted of 50.4-54Gy of radiation and concomitant 5FU-based chemotherapy. Patients were clinically reassessed for tumor response at least 12 weeks from nCRT completion using digital rectal examination, rigid proctoscopy, pelvic MRI and CEA levels [6]. Patients with evidence of residual disease (incomplete clinical response) such as residual ulcers, irregularity, mass or stenosis at clinical examination were referred to immediate radical surgery. Likewise, patients with radiological evidence of residual disease including the presence of nodal metastases or residual primary cancer were also referred to radical surgery. Patients with clinical, endoscopic or radiological evidence of cCR were recommended no immediate radical surgery and were enrolled in a strict follow-up program (Watch & Wait strategy) [5].

### Groups for comparison

Based on chronological order, the first patients matching our criteria were included in the training set (27 samples) and the samples collected later on were included in the validation set (16 samples). Patients were grouped according to response to nCRT based on clinical and pathological findings. The complete responders group included patients with clinical evidence of complete response (cCR) sustained for at least 24 months of follow-up and patients with complete pathological response (pCR) who underwent radical surgery due to inability to rule out residual disease. The incomplete responders group included patients with significant residual disease detected after pathological examination of the resected specimen, including ypT2-4 or ypN+ and >10% residual cancer cells (tumor regression grades - TRG0-2 according to Dworak’s classification [14]. Therefore, patients with incomplete but with “near-complete” response including ypT1N0 or ≤10% residual cancer cells (TRG3) were excluded from the study. Differential miRNA expression analyses were performed comparing the complete to incomplete responders.

### miRNA sequencing analysis

Total RNA was extracted with Trizol reagent (Invitrogen), and RNA quality was evaluated on 2100 Bioanalyzer (Agilent). All samples had RNA integrity number (RIN) above 6. Total RNA (10 μg) was enriched for small RNAs (up to 250 bp) using PureLink™ miRNA Isolation Kit (Invitrogen). miRNA libraries were prepared using SOLiD™ Total RNA-Seq Kit (Life Technologies), according to the manufacturer’s recommendations and were sequenced on SOLiD4 (Life Technologies) in 35 bp single read runs.

Sequencing data was analyzed using the CLC Genomics Workbench 5.1 software (CLC Bio). Initially, sequencing adapters were trimmed from sequencing reads. The remaining sequences were mapped against the human miRNA database, miRBase (www.mirbase.org, release 18) [15,16], allowing one mismatch. At last, known mature miRNAs were annotated according to miRBase and the number of sequences for each miRNA was used for differential expression analysis. Normalization was done by dividing the number of sequences of a certain miRNA by the total number of miRNA mapped sequences for a given sample and multiplying by one million. This resulted in a value corresponding to the number of sequences (counts) per million (cpm) for each miRNA. After normalization, low expressed miRNAs were filtered from further analysis to increase detection power of the statistic tests. Only miRNAs with a minimum of 20 cpm in at least seven samples were kept in the analyses (Additional file 1: Table S1).

Differential expression between the two groups of patients was performed with EdgeR (version 2.6.7) [17] available on Bioconductor 2.10 [18]. P-values were adjusted for multiple testing using the Benjamini and Hochberg method to control the false discovery rate (FDR) [19]. Only miRNAs with FDR under 0.05 were considered as differently expressed.

### Clustering and response prediction analysis

Hierarchical clustering and bootstrap statistical analysis were performed using the functions ‘heatmap’ and ‘pvclust’ available in the R statistical software (version 3.0.1). These analyzes were based on Euclidean distance and average linkage clustering using cpm normalized data. Principal Component Analysis (PCA) was performed using the Matlab language function ‘princomp’ (version R2009b). PCA was carried out by eigenvalue decomposition of the covariance matrix after mean centering the data matrix for each attribute. The first principal component (*i.e.*, the direction along which miRNAs expression show the largest variance) and the second principal component (*i.e.*, the next best direction uncorrelated with first one) were retrieved and plotted to illustrate similarities between samples.

Receiver operating characteristic (ROC) curve for each miRNA was generated using cpm values obtained from RNA-Seq data, using GraphPad Prism software (version 4.03). To determine accuracy to predict nCRT, the area under the ROC curve (AUC) was calculated. The cutoff expression values (cpm) were defined in order to maximize the number of correctly classified samples. Finally, sensitivity and specificity based on this cutoff were calculated.

### miR-21-5p putative target genes

We initially used TargetScan software (www.targetscan.org) to search for predicted miR-21-5p targets [20]. We considered only conserved sites in conserved target genes. Using whole transcriptome sequencing data from 19 of the primary rectal tumors used in this study (unpublished data), we searched for the expression of predicted targets that were inversely correlated (Pearson correlation) to miR-21-5p expression using GraphPad Prism software (version 4.03).

Further exploring whole transcriptome data, SATB1 gene expression was compared between incomplete responders (n = 13) and complete responders (n = 6). Mann–Whitney test was performed using GraphPad Prism software (version 4.03).

### Validation of miR-21-5p and SATB1 expression by qPCR

miR-21-5p and SATB1 gene expression was evaluated through qPCR in 10 and 11 samples, respectively (the samples that were used for validation of SATB1 were the same as the samples for miR-21-5p plus another sample from an incomplete responder that still had RNA available). For miR-21-5p, the QIAGEN miScript PCR system was used. cDNA was generated with miScript II RT kit (QIAGEN) and 3 ng were used as template in each qPCR reaction. miR-21-5p miScript primer assay and miScript SYBR Green PCR kit were also used. Reactions were performed in triplicates using 7900HT Fast System (Applied Biosystems). Based on our miRNA-Seq data, we chose miR140-5p and miR-224-5p to be used for normalization of qPCR experiments since they were most stably expressed miRNAs among our samples [21,22].

SATB1 gene expression was evaluated with GoTaq® qPCR Master Mix (Promega) using the following primers: SATB1Fw: GGTACAAACATTTCAAGAAGAC and SATB1Rev: CATGATTGGCGCCTTGCT. PUM1 and HMBS genes were used for normalization (PUM1Fw: TGTACTTACGAAGAGTTGCGATGTG PUM1Rev: CCAGGCCAGCGGAAGAT; HMBSFw: GGCAATGCGGCTGCAA HMBSRev: GGGTACCCACGCGAATCAC).

Relative expression was calculated based on ΔΔCT method using a colorectal cancer cell line (HCT116) as reference sample [23]. Mann–Whitney test was performed using GraphPad Prism software (version 4.03).

### Clonogenic assays

Colorectal tumor cell lines, HCT116 and SW480, were obtained from American Type Culture Collection (ATCC), and cultured following their recommendations.

Cells were plated at a low density (500 cells/well) in 4 wells plates (1.9 cm^2^ well area) and transfections were carried out with Lipofectamine® RNAiMAX Transfection Reagent (Invitrogen). HCT116 were transiently transfected with 30 nM mirVana™ miRNA Inhibitors (hsa-miR-21-5p or inhibitor control) and SW480 were transfected with 10 nM mirVana™ miRNA Mimics (hsa-miR-21-5p or mimic control) (Life Technologies). 24 h after transfection, cells were treated with 5 μM 5-fluorouracil (5-FU, Libbs) and irradiated with 1 Gy dose for HCT116 and 2Gy for SW480 using GammaCell 3000 equipment (Elan). After 12 days, colony formation was quantified. Cells were fixed with formaldehyde, stained with crystal violet and air-dried. For colony quantification, the dye from stained cells was solubilised with 10% acetic acid and absorbance was measured at 595 nm using Infinite 200 PRO (Tecan Group Ltd). Two independent experiments were performed in quadruplicates for each cell line.

## Results

### Training and validation sets of patients

Clinical and demographics features of all 43 patients included in this study are summarized in Table 1. Overall, 27 patients were analyzed in the training set, including 7 complete responders and 20 incomplete responders. In addition, 16 patients were analyzed as a validation set, being 7 complete responders, 5 incomplete responders and 4 patients that initially presented cCR but developed early local recurrence (within the first 16 months of follow-up after nCRT).

**Table 1:** **Clinical and demographics data of patients included in the study**

	**Training set**			**Validation set**			
	**Complete responders**	**Incomplete responders**	**p**	**Complete responders**	**Incomplete responders**	**Recurrence**	**p**
**N**	7	20		7	5	4	
**Age (years)**	58.9 ± 10.9	54.2 ± 13.7	ns	60.4 ± 13.6	59.6 ± 14.5	69.3 ± 15.0	ns
**Gender (M/F)**	2/5	9/11	ns	3/4	3/2	3/1	ns
**Distance anal verge (cm)**	4.0 ± 2.6	4.3 ± 2.2	ns	4.6 ± 2.4	2.2 ± 2.8	4 ± 0.8	ns
**Tumor size (cm)**	4.4 ± 0.8	4.6 ± 1.5	ns	3.6 ± 0.9	5 ± 1.9	5 ± 1.4	ns
**Initial staging**			ns				ns
T2	0	2		4	2	0	
T3	7	17		2	2	4	
T4	0	1		1	1	0	
N0	4	7		2	2	2	
N+	3	13		5	3	2	
**Staging after CRT**							
cCR	3	-		6	-	4	
pCR	4	-		1	-	-	
ypT2	-	6		-	1	-	
ypT3	-	13		-	4	-	
ypT4	-	1		-	0	-	
ypN0	-	12		-	1	-	
ypN+	-	8		-	2	-	
ypNx	-	0		-	2	-	

### miRNA differential expression to predict nCRT

Primary tumor biopsies were collected before nCRT and used for small RNA sequencing. On average, 37 million sequences were generated for each sample. After mapping and annotation, 711 mature miRNAs were detected per sample. The number of sequences for each specific miRNA was used to estimate its expression, and low expressed miRNAs were removed in order to increase detection power of statistic tests. Finally, we performed differential expression analysis using, on average, 330 mature miRNAs for each sample (Additional file 1: Table S1).

The first approach to identify molecular markers capable of predicting tumor response to nCRT was to perform differential miRNA expression analysis using the 27 samples from the training set. Comparison between complete and incomplete responders showed 36 differently expressed miRNAs (p < 0.05). In order to apply a more stringent control over false positives, p-values were adjusted for multiple testing, which controls false discovery rate (FDR) [19]. Thus, 4 miRNAs had a FDR < 0.05 and were considered as reliable differentially expressed when comparing complete to incomplete responders (Table 2). Three miRNAs were overexpressed in complete responders (miR-21-5p, miR-1246, and miR-1290-3p) and one, miR-205-5p, was overexpressed in incomplete responders.

**Table 2:** **Differentially expressed miRNAs**

**miRNA**	**Complete responders mean expression (cpm)**	**Incomplete responders mean expression (cpm)**	**Fold change**	**P-value**	**FDR**
miR-205-5p	3.67	2,099.32	0.002	6.21E-06	0.002
miR-21-5p	106,020.55	31,438.53	3.372	1.99E-04	0.028
miR-1290-3p	115.14	30.02	3.835	2.65E-04	0.028
miR-1246	65.58	19.60	3.346	3.36E-04	0.028

miRNAs mean expression is shown for each response group to nCRT and fold change of complete responders relative to incomplete responders. Statistical significance can be verified by P-values and false discovery rate (FDR).

**Figure 1:**
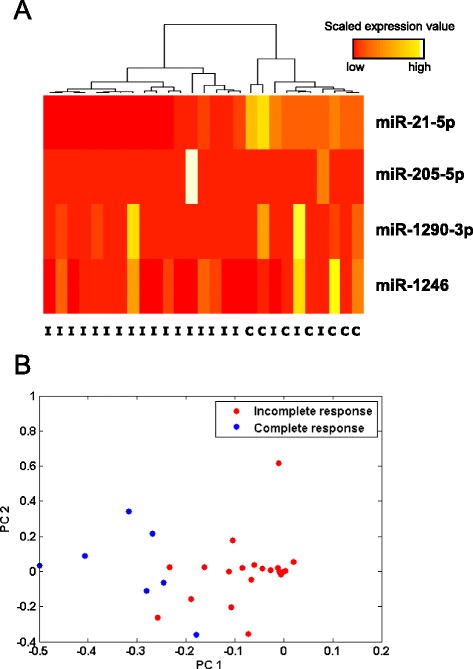
**Patients’ clustering based on the expression of 4 differently expressed miRNAs. A)** Hierarchical clustering heatmap for incomplete (I) and complete responders (C). **B)** PCA was conducted for 27 samples and 4 variables (differently expressed miRNAs). Blue dots: complete responders; red dots: incomplete responders.

After determining differentially expressed miRNAs, we evaluated their efficiency in discriminating complete responders from incomplete responders’ patients. First, we performed hierarchical clustering analysis which resulted in two major groups, one containing only incomplete responders and the other one with all complete responders plus 3 incomplete responders (Figure 1A). Second, the expression level (given in count per million, cpm) of the 4 differentially expressed miRNAs was used to perform Principle Component Analysis (PCA). This analysis revealed good separation between complete and incomplete responders (Figure 1B). Consistent with the hierarchical clustering analysis, the same 3 incomplete responders showed greater similarity with patients from the complete responders group.

### miRNAs as potential predictive markers

**Figure 2:**
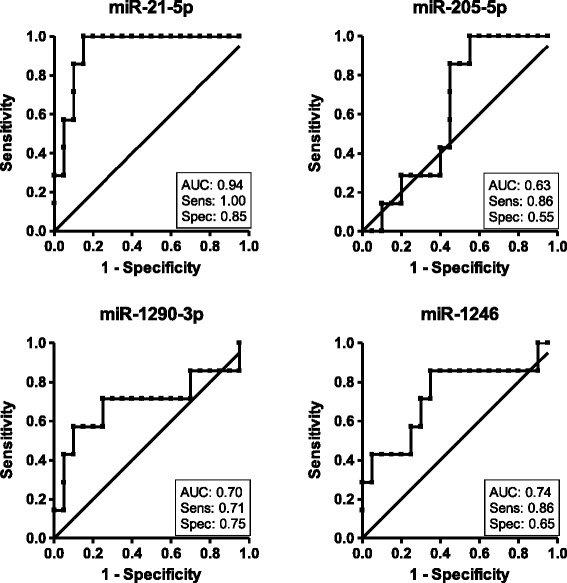
**Accuracy to predict nCRT based on the expression of differently expressed miRNAs.** ROC curves were built based on miRNA expression determined by RNA-Seq. Area under the curve (AUC), sensitivity (Sens) and specificity (Spec) were calculated for each miRNA.

We have also investigated the potential of each of the 4 differently expressed miRNAs to be individually used as a predictive biomarker. In order to estimate the efficiency on correctly classifying patients according to treatment response, we calculated the area under the ROC curve (AUC) for each miRNA. We found an AUC of 0.94, 0.74, 0.70, and 0.63 for miR-21-5p, miR-1246, miR-1290-3p, and miR-205-5p respectively (Figure 2). Also, the expression cutoff point was determined for each miRNA to maximize sensitivity and specificity. Considering the normalized expression values (in cpm) obtained by RNA-Seq, the established cutoff for each miRNA resulted in a sensitivity and specificity of 100% and 85% for miR-21-5p, 86% and 65% for miR-1246, 71% and 75% for miR-1290-3p, and 86% and 55% for miR-205-5p, respectively (Figure 2). These results suggested miR-21-5p as the best putative predictive marker.

### Validation of miRNA differential expression in independent samples

In order to verify whether the differential expression of these miRNAs was not limited to the training set of samples, we have also analyzed a validation set of samples, including 7 complete responders and 5 incomplete responders. miRNAs libraries from these primary tumors were sequenced and global differential expression analysis between complete and incomplete responders was performed. Thus, we found miR-21-5p as a common differently expressed miRNA (p-value < 0.01). The other 3 miRNAs did not show differential expression in the validation set and therefore, were not used in the following analyses.

**Figure 3:**
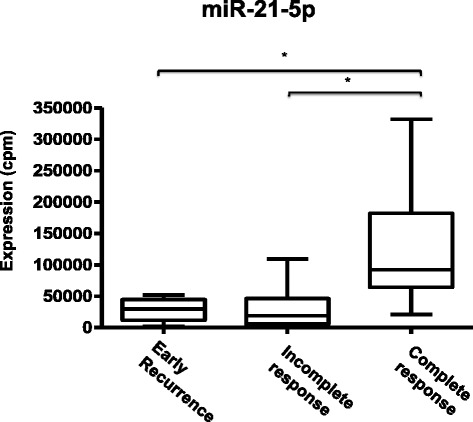
**miR-21-5p expression in all samples included in the study.** Patients were divided in 3 groups according to nCRT response: patients with initial cCR followed by early local recurrence (n = 4), incomplete responders (n = 25), and complete responders (n = 14). miR-21-5p expression is given as normalized values (cpm) from RNA-Seq data. Statistical analysis was performed with EdgeR (*FDR < 0.05).

To further validate miR-21-5p as a predictive marker for nCRT, we also analyzed its expression in samples from patients that initially presented cCR to nCRT and were spared from immediate surgery but during follow up visits, presented early local recurrence requiring salvage resection (named here as early recurrence group). These patients most definitely had clinically undetectable residual disease after nCRT. Noteworthy, miR-21-5p expression pattern in these patients was very similar to incomplete responders (Figure 3). More importantly, miR-21-5p expression in these two groups of patients (incomplete responders and early recurrence group) was significantly lower than the complete responders group (p-value <0.01). These results indicated that although physicians could not distinguish between patients who presented “bona fide” complete response and patients who initially presented complete clinical response but faced early local recurrence, they have tumors with important molecular differences, including miR-21-5p expression.

In addition, we determined the overall predictive power of miR-21-5p expression using the cutoff established on the ROC curve for the training set (70,000 cpm) and all the samples included in the study (from training and validation set). For this estimation, we considered the early recurrence group as incomplete responders. Thus, miR-21-5p expression presented 78.5% (11/14) sensitivity and 86% (25/29) specificity to identify patients with complete response to nCRT. Similarly, miR-21-5p expression also showed good positive (73%, 11/15) and negative predictive values (89%, 25/28).

Finally, we have also validated the miR-21-5p differential expression by qPCR, a more easily and cheaper implemented method compared to deep-sequencing. Since our samples were all pre-treatment rectal tumor biopsies, they yielded a limited amount of RNA usually completely used for miRNA library construction. Therefore, for qPCR validation, we only tested 10 samples that still had RNA available (3 complete responders and 7 incomplete responders). The higher miR-21-5p expression in complete responders was once again validated (Additional file 2: Figure S1). However, possibly due to the small number of samples tested, this difference was not statistically significant (p-value = 0.067).

We have also investigated whether miR-21-5p expression was associated with clinical and pathological parameters. In order to do that, we categorized samples into two groups: high miR-21-5p expression (expression above the established cutoff, 70,000 cpm) and low miR-21-5p expression. No statistically significant association was found between miR-21-5p expression and any of the parameters analyzed, such as age, gender, tumor size, tumor distance from anal verge, and initial T and N staging (Additional file 3: Table S2).

### Potential role of miR-21-5p on treatment response

**Figure 4:**
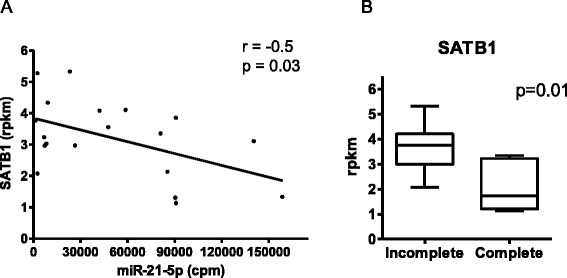
**Expression analysis of** ***SATB1*** **gene. A)** Correlation between SATB1 and miR-21-5p expression, measured by RNA-Seq, in the same primary rectal tumors. r: Pearson correlation factor. **B)** SATB1 expression according to nCRT response: incomplete responders (n = 13), and complete responders (n = 6). SATB1 expression is given as normalized values (rpkm). Mann–Whitney test performed.

In order to investigate whether miR-21-5p expression is directly involved in treatment response, we analyzed the expression pattern of its putative target genes using whole transcriptome RNA-Seq data from 19 samples of the training set patients (6 complete responders and 13 incomplete responders – unpublished data). Using TargetScan software [20], we found 307 predicted miR-21-5p targets genes with high prediction score. Among them, we were able to detect the expression (measured in reads per kilobase of transcript per million reads mapped, RPKM) for 249 genes in the 19 patients. Considering the negative regulation of miRNAs over their target genes’ expression, we searched for predicted targets for which the expression pattern was inversely correlated to miR-21-5p expression. Using this approach, we only found a significant negative correlation (*r* = −0.5 and p-value = 0.03) for *SATB1* gene (Figure 4A and Additional file 4: Table S3). This is a global gene regulator that has been reported to confer malignant behavior and multidrug resistance (MDR), as well as being associated with poor prognosis in several cancer types, including rectal cancer [24-26]. Even though other authors have shown that miR-21-5p can regulate the expression of other target genes such as PTEN, MSH2, Cdc25A, SPRY2 and PDCD4, and therefore be associated with worse response to therapy in other tumor types, we did not observe a significant inverse correlation between the expression of these target genes and of miR-21-5p in our samples (Additional file 3: Table S4) [27-31].

**Figure 5:**
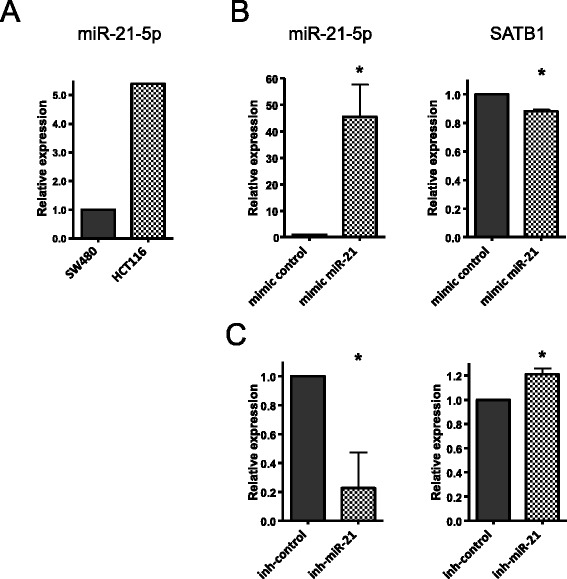
**Manipulation of miR-21-5p expression negatively correlates with SATB1 mRNA expression. A)** Evaluation of miR-21-5p expression by qPCR shows that HCT116 and SW480 cells express different levels. **B)** miR-21-5p and SATB1 mRNA expression were evaluated by qRT-PCR 24 hours after transient transfection of SW480 cell line with miR-21-5p mimic or mimic control; **C)** miR-21-5p and SATB1 mRNA expression was evaluated 24 hours after transient transfection of HCT116 cell line with miR-21-5p inhibitor or inhibitor control. Bars represent mean expression relative to control and normalized by RNU6b (for miR-21-5p) and HMBS (for SATB1). Error bars represent standard deviation for quadruplicates in 2 independent experiments. *P < 0.005 for Student’s *t*-test.

It has already been shown that miR-21-5p can negatively regulate SATB1 expression [32,33]. Indeed, we have confirmed such regulation *in vitro* by transiently manipulating miR-21-5p expression in 2 different colorectal cancer cell lines. HCT116 and SW480 cells were chosen because they differ significantly on miR-21-5p expression levels: 5-fold higher in HCT116 cells (Figure 5A). Thus, we have transfected SW480 cells with miR-21-5p mimic and observed by qPCR a reduction on SATB1 mRNA expression (Figure 5B). Accordingly, we have knocked-down miR-21-5p expression in HCT116 cancer cells and observed an increased expression of SATB1 (Figure 5C). These results confirmed the negative regulation of miR-21-5p on SATB1 expression and corroborated the negative correlation of their expression observed in our patients’ samples.

Further exploring the whole transcriptome data from these 19 patients, we found a significantly higher SATB1 expression in incomplete responders compared to complete responders, according to our hypothesis (p-value = 0.01) (Figure 4B). In addition, we have also evaluated SATB1 expression by qPCR in 11 primary tumors (3 complete and 8 incomplete responders). A higher SATB1 expression in incomplete responders was once again observed. However, similar to what we observed for miR-21-5p, this difference was not statistically significant (p-value = 0.13) (Additional file 2: Figure S2). These results suggest SATB1 as a potential candidate for functional studies, which could elucidate the possible direct involvement of miR-21-5p in response to nCRT in rectal cancer patients.

### miR-21-5p and SATB1 expression in colorectal cancer cells is directly involved with treatment response

Further exploring the involvement of miR-21-5p on response to nCRT in rectal cancer patients we have challenged HCT116 and SW480 colorectal cancer cell lines to a similar treatment *in vitro*. In order to properly evaluate the role of miR-21-5p, we have transiently manipulated its expression in both of them and performed clonogenic assays to verify whether there were changes on their sensitivity to the chemoradiation treatment. Experiments were performed with cells cultured under control conditions as well as submitted to one dose of radiation combined with 5-FU treatment (see details in Methods).

**Figure 6:**
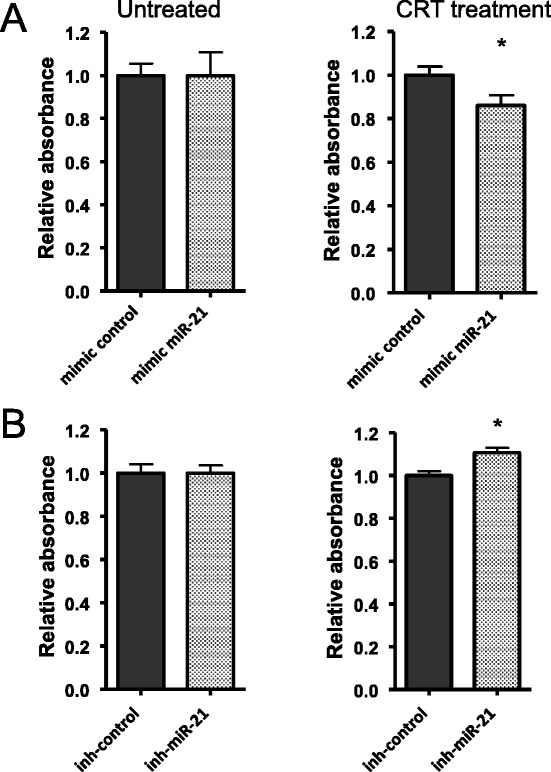
**Manipulation of miR-21-5p expression alters response to chemoradiation** ***in vitro*** **. A)** Quantification of colony formation assays for SW480 cell line transfected with miR-21-5p mimic or mimic control. **B)** Quantification of colony formation assays for HCT116 cell line transfected with miR-21-5p inhibitor or inhibitor control. Cells were grown under control conditions or submitted to CRT treatment, which consisted of 5 μM 5-FU and a single dose of 2Gy **(A)** or 1Gy **(B)** radiation. Graphs show relative absorbance of colonies stained with crystal violet, normalized to untreated controls. All values are presented as means ± s.d. relative to mimic or inhibitor controls from two independent experiments with quadruplicates. *P < 0.005 for Student’s *t*-test.

Firstly, SW480 cells were transiently transfected with miR-21-5p mimic and mimic control, and submitted to the clonogenic assay. As expected, miR-21-5p increased expression resulted in decreased SATB1 expression and also turned SW480 cells more sensitive to chemoradiation treatment (Figure 6A). Similarly, HCT116 cells, which express higher levels of miR-21-5p, were transiently transfected with mir-21-5p inhibitor and inhibitor control, and submitted to the clonogenic assay. Inhibition of miR-21-5p expression resulted in an increased SATB1 expression and decreased sensitivity to chemoradiation treatment (Figura 6B). According to our findings in patients, these results suggest that, through regulation of SATB1 expression, miR-21-5p is directly involved on response to chemoradiotherapy.

## Discussion

Standard treatment of locally advanced rectal cancer may include nCRT followed by radical surgery in most patients. However, the observation of pCR in up to 42% of patients undergoing nCRT has challenged the role of radical surgery in this setting and alternative treatment strategies for these patients have been suggested [4,5,34]. Identification of these patients by clinical and imaging assessments may be very difficult and remains restricted to specific dedicated centers. Therefore, most of these patients still undergo potentially unnecessary radical surgery including total mesorectal excision. Molecular markers capable of accurately predicting tumor response to nCRT would be of great clinical significance since these patients could be spared from radical surgery and from morbidities associated with this procedure.

Previous studies searching for predictive molecular markers in rectal cancer patients have used microarray and qPCR to evaluate mRNA or miRNA profiling [35-42]. However, patients’ response classification varied considerably among these studies. The “Responders” group frequently included complete pathological responders and partial responders (based on T-level downstaging or tumor regression grades). In addition, although it has been shown that pCR rates may considerably increase up to 12 weeks after nCRT, most of these studies have assessed tumor response at considerably short intervals from nCRT (most frequently after 6 weeks) [35,37,38,43]. Thus, comparison between studies may be quite difficult and rather inconclusive. Regarding miRNA profiling, three recent studies have identified signatures with good accuracy to predict nCRT response [40-42]. However, none of them validated their signatures in an independent group of patients. Importantly, comparing the molecular signatures, there is no overlapping miRNA among them, suggesting that these signatures might be characteristic of the samples used in each study.

To the best of our knowledge, this is the first study to perform global miRNA sequencing followed by differential expression analysis of primary rectal tumor samples, and to use, as complete response group, samples from patients with pCR as well as with sustained cCR to nCRT. In addition, the exclusion of patients with “near-complete” responses rather than including them in complete or incomplete responder groups allowed us to compare only patients/tumors with significant clinical discrepancies in their outcomes (response to nCRT), favoring the identification of molecular differences. Using this approach, we identified 4 differently expressed miRNAs between complete and incomplete responders in the training set. Based on their expression and by performing two different analyses (hierarchical clustering and PCA), samples could be clustered into two distinct groups. Importantly, in all of them, all 7 complete responders were clustered together. The predictive power of each miRNA was also estimated through ROC curves and miR-21-5p showed the best accuracy (AUC = 0.94).

Due to clinical and molecular heterogeneity of tumor samples, we expect that predictive markers found in one group of samples might not be valid for a different group of samples. Thus, miRNA differential expression was further evaluated in additional samples. Among the 4 miRNAs, miR-21-5p maintained a significant difference in expression levels, being overexpressed in patients with complete response to nCRT, and showing the potential of this miRNA to be used as a predictive marker. Interestingly, in all patients with initial cCR followed by early local recurrence, miR-21-5p showed an incomplete response expression pattern. Thus, if miR-21-5p expression was used to help physicians to decide whether to operate these patients or not, they would have been correctly submitted to surgery immediately after the resting period post-nCRT.

When used as a predictive marker on all samples included in the study, miR-21-5p expression showed high sensitivity (78.5%) and specificity (86%) on the identification of complete responders. Among complete responders, it is important to note that miR-21-5p expression was not significantly different between pCR and cCR. Importantly, considering only pCR patients, 4 out of 5 (80%) were correctly predicted based on miR-21-5p expression. These patients were submitted to surgery due to inability to rule out residual disease by clinical and radiological exams, suggesting again that a high percentage of patients could benefit from such biomarker and be spared from radical surgery. Although we have studied a limited number of samples, this is the first study that has used a validation set of samples to confirm the differential expression observed in a training set. Thus, miR-21-5p emerges as a promising predictive marker to nCRT that still needs to be further validated in a larger cohort.

miR-21-5p is overexpressed in different tumor types, including colorectal cancer (CRC), possibly acting as an oncogenic miRNA with important roles in cell proliferation, apoptosis and invasion [44-46]. Therefore, miR-21-5p has been considered a promising diagnostic/prognostic biomarker [47-49]. However, the literature is controversial regarding miR-21-5p expression and treatment response. Although several *in vitro* studies using CRC cell lines have shown that overexpression of miR-21-5p induces resistance to chemotherapy [30,31,50], few studies evaluated miR-21-5p expression in CRC primary tumors and in the context of radiotherapy. In one of these studies, patients treated with adjuvant chemotherapy, high miR-21-5p expression predicted worse overall survival, suggesting an association between high miR-21-5p expression and poor therapeutic outcome [51]. However, comparison between these results and ours is not straightforward, since they investigated miR-21-5p expression in the context of colon adenocarcinoma, adjuvant chemotherapy and long-term survival outcomes. All of these discrepancies lead to inherent limitations in comparisons between studies and possibly yielding controversial findings.

In one of the few studies with rectal cancer patients submitted to nCRT, miR-21 expression was evaluated in macrodissected tumor tissue before and after treatment, and in normal rectal tissue from resection specimen [52]. There was no significant difference on miR-21 expression level between pre and post-treatment tumor samples as well as between post-treatment tumor and normal tissues.

miRNAs’ functions are usually inferred through the identification of their target genes. Considering the negative regulation imposed by miRNAs it can be assumed that they perform an opposite role of their targets. Searching for the expression of miR-21-5p targets in our samples, we found an interesting negative correlation for *SATB1* gene and miR-21-5p expression. As expected, when we compared SATB1 expression among complete and incomplete responders, a significantly higher expression was observed in incomplete responders. SATB1 is a chromatin modifier and it has also been shown to be a MDR gene in several cancer types [25,53,54].

As mentioned, although other authors have shown that miR-21-5p can regulate the expression of other target genes such as PTEN and MSH2, and therefore be associated with worse response to therapy, we did not observe an inverse correlation between the expression of any these target genes and of miR-21-5p in our samples. Therefore, as miRNAs’ function can be tissue specific, we hypothesize that, in the context of rectal cancer samples, miR-21-5p might not be involved on the regulation of these target genes.

Finally, we have also performed functional experiments with two different cancer cell lines. We not only confirmed the regulation of SATB1 expression by miR-21-5p but more importantly, we have confirmed that miR-21-5p and SATB1 play a direct role in response to *in vitro* chemoradiation.

## Conclusions

We have identified miR-21-5p as a promising predictive marker for response to nCRT in rectal cancer patients. Patients with cCR based on very stringent criteria and high levels of miR-21-5p expression may be ideal candidates for alternative treatment strategies to radical surgery including a watch and wait approach. Further validation of these findings in larger cohorts may lead to the inclusion of this miRNA-based test in standard workup of patients prior to nCRT and help in clinical decision-making for patients with apparent cCR.

## Additional files

Additional file 1: Table S1. Expression of miRNAs used for differential expression analyses among complete and incomplete responders. Samples from 1 to 14 represent complete responders patients, 15 to 39 represent incomplete responders patients, and 40 to 43 represent patients with early local recurrence. Normalized expression values are given in count per million (cpm). Additional file 2:
**Figure S1.** Evaluation of miR-21-5p by qPCR on primary tumor samples. Samples still available included 7 incomplete and 3 complete responders to nCRT. A colorectal cancer cell line (HCT116) was used as reference sample and the relative expression was calculated based on ΔΔCT method using miR-140-5p and miR-224-5p expression for normalization. Mann–Whitney test performed. **Figure S2.** Evaluation of SATB1 gene expression by qPCR on primary tumor samples. Samples from 3 complete and 8 incomplete responders were used for SATB1 validation. A colorectal cancer cell line (HCT116) was used as reference sample and the relative expression was calculated based on ΔΔCT method using PUM1 and HMBS gene expression for normalization. Mann–Whitney test performed. Additional file 3: Tables S2. Patients’ clinical characteristics distribution according to miR-21-5p expression level (70,000 cpm cutoff established based on the training group). **Table S4.** Expression of miR-21-5p and 5 of its published target genes in rectal cancer samples. Pearson correlation was evaluated and only SATB1 gene presented a significant inversed correlation (r = −0.5, p = 0.03). Additional file 4: Table S3. Pearson correlation between the expression of miR-21-5p and all putative target genes in rectal cancer samples. Only SATB1 gene presented a significant inversed correlation (r = −0.5, p = 0.03).
