# Supplementary material for: Overexpression of miR-21-5p as a predictive marker for complete tumor regression to neoadjuvant chemoradiotherapy in rectal cancer patients
Source: BMC Med Genomics. 2014 Dec 11;7:68. doi: 10.1186/s12920-014-0068-7 (PMC4279677; doi:10.1186/s12920-014-0068-7)
Supplement: Additional file 2: — Figure S1. Evaluation of miR-21-5p by qPCR on primary tumor samples. Samples still available included 7 incomplete and 3 complete responders to nCRT. A colorectal cancer cell line (HCT116) was used as reference sample and the relative expression was calculated based on ΔΔCT method using miR-140-5p and miR-224-5p expression for normalization. Mann–Whitney test performed. Figure S2. Evaluation of SATB1 gene expression by qPCR on primary tumor samples. Samples from 3 complete and 8 incomplete responders were used for SATB1 validation. A colorectal cancer cell line (HCT116) was used as reference sample and the relative expression was calculated based on ΔΔCT method using PUM1 and HMBS gene expression for normalization. Mann–Whitney test performed. [file 12920_2014_68_MOESM2_ESM.pdf]

**Additional file 4 (.pdf): Supplementary Figures 1 and 2**

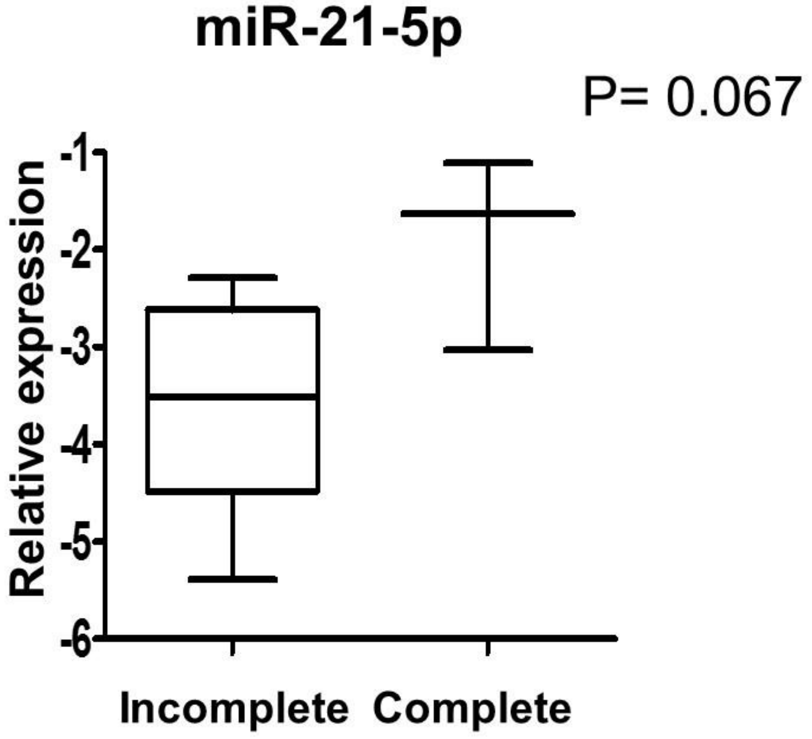


**Figure S1– Evaluation of miR-21-5p by qPCR on primary tumor samples.** Samples still available included 7 incomplete and 3 complete responders to nCRT. A colorectal cancer cell line (HCT116) was used as reference sample and the relative expression was calculated based on ΔΔCT method using miR-140-5p and miR-224-5p expression for normalization. Mann-Whitney test performed.

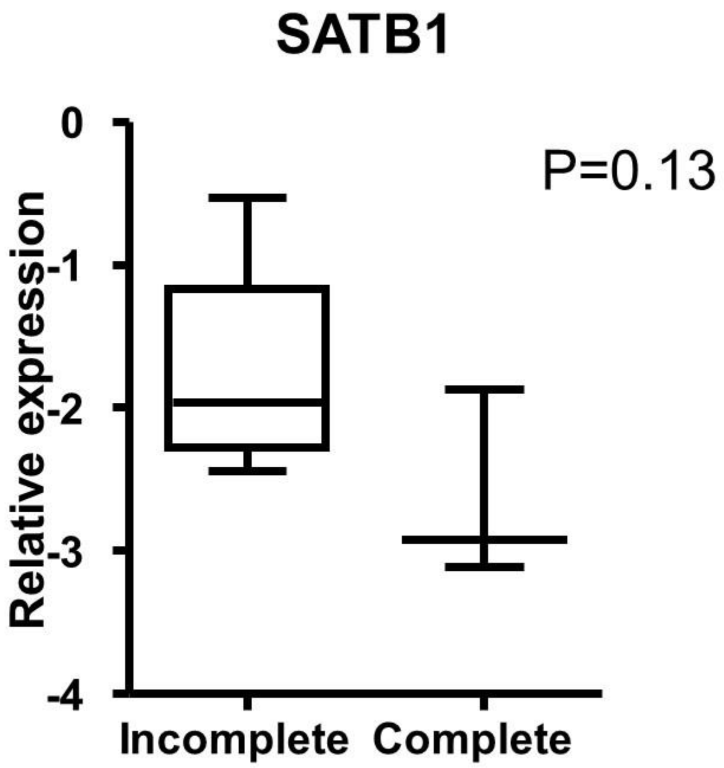


**Figure S2– Evaluation of SATB1 gene expression by qPCR on primary tumor samples.**

Samples from 3 complete and 8 incomplete responders were used for SATB1 validation. A colorectal cancer cell line (HCT116) was used as reference sample and the relative expression was calculated based on ΔΔCT method using PUM1 and HMBS gene expression for normalization. Mann-Whitney test performed.
