# Supplementary material for: Overexpression of miR-21-5p as a predictive marker for complete tumor regression to neoadjuvant chemoradiotherapy in rectal cancer patients
Source: BMC Med Genomics. 2014 Dec 11;7:68. doi: 10.1186/s12920-014-0068-7 (PMC4279677; doi:10.1186/s12920-014-0068-7)
Supplement: Additional file 3: Tables S2. — Patients’ clinical characteristics distribution according to miR-21-5p expression level (70,000 cpm cutoff established based on the training group). Table S4. Expression of miR-21-5p and 5 of its published target genes in rectal cancer samples. Pearson correlation was evaluated and only SATB1 gene presented a significant inversed correlation (r = −0.5, p = 0.03). [file 12920_2014_68_MOESM3_ESM.pdf]

Additional file 2 (.pdf) – Supplementary Tables 2 and 4

Table S2: Patients' clinical characteristics distribution according to miR-21-5p expression level (70,000 cpm cutoff established based on the training group).

**Characteristic**	**miR-21-5p-expression**		**p-value**
	**Low**	**High**	
**N**	28	15	
**Age (years)**	58.3 ± 14.2	57.4 ± 12.9	0.835 [1]
**Gender (M/F)**	15/13	8/7	1.000 [2]
**Distance anal verge (cm)**	4.3 ± 2.1	3.5 ± 2.5	0.322 [1]
**Tumor size (cm)**	4.6 ± 1.6	4.3 ± 0.8	0.391 [1]
**Initial T staging**			0.257 [3]
T1	1	0	
T2	3	4	
T3	23	9	
T4	1	2	
**Initial N staging**			0.528 [2]
N0	10	7	
N+	18	8	
**Response after nCRT**			<0.0001 [2]
Complete	3	11	
Incomplete	25	4	

[1] T test. - [2] Fisher exact test. – [3] Chi square test.

Table S4: Expression of miR-21-5p and 5 of its published target genes in rectal cancer samples. Pearson correlation was evaluated and only SATB1 gene presented a significant inversed correlation (r=-0.5, p = 0.03).

Sample	miR-21-5p	SATB1	CDC25A	PDCD4	PTEN	MSH2	SPRY2
2	140,635.00	3.10	3.20	67.12	14.99	10.65	11.57
6	81,249.54	3.35	3.35	21.01	11.65	7.68	13.20
7	90,589.45	1.30	3.87	11.62	10.47	12.06	6.10
8	91,067.92	1.12	8.12	7.66	12.94	26.85	12.40
11	158,834.71	1.33	3.57	6.04	9.43	9.36	12.04
14	85,609.41	2.12	1.86	13.07	14.93	5.00	13.16
16	42,314.23	4.08	1.39	20.20	13.53	11.45	20.04
17	2,722.60	2.07	4.98	15.21	8.81	13.13	10.84
20	58,916.61	4.10	9.42	13.25	11.46	17.04	13.97
22	2,720.95	5.27	2.36	40.85	14.72	9.61	20.13
23	47,877.24	3.55	6.47	6.00	8.58	19.33	13.63
25	6,978.22	3.23	2.43	8.51	6.76	10.45	22.16
26	9,335.28	4.33	2.09	7.06	9.97	7.54	6.69
27	26,630.82	2.96	3.22	13.61	8.35	8.38	12.28
30	8,400.31	3.02	2.47	43.86	18.89	22.61	10.86
31	23,401.71	5.32	3.00	11.98	10.95	8.55	13.71
32	91,005.47	3.85	3.83	8.14	8.31	10.58	9.79
35	7,332.10	2.96	4.42	17.16	8.56	22.18	14.47
36	1,367.17	3.76	1.79	10.56	11.29	6.45	14.64
	**Pearson correlation r**	**-0.502**	**0.20**	**0.10**	**0.10**	**-0.06**	**-0.32**
	**P value**	**0.0285**	**0.40**	**0.67**	**0.70**	**0.80**	**0.18**
